# Study on the effect and mechanism of Qufushengji hydrogel based on network pharmacology and molecular docking technology in treating phlebitis

**DOI:** 10.1097/MD.0000000000047427

**Published:** 2026-02-06

**Authors:** Zhe Meng, Shuangxin Zhang, Qirui Zhang, Yijia Lin, Juncai Li, Longjie Wei, Bonolo William, Xiuling Zhou

**Affiliations:** a School of Nursing, Changchun University of Chinese Medicine, Changchun, China.

**Keywords:** molecular docking, network pharmacology, pathway analysis, phlebitis, traditional Chinese medicine

## Abstract

Intravenous therapy often triggers phlebitis as an adverse effect, and there are limitations in modern medical treatments. The aim of this study was to investigate the mechanism of action of Qufushengji hydrogel, a novel topical preparation of traditional Chinese medicine, in the treatment of phlebitis. Based on network pharmacology and molecular docking technology, we screened the active ingredients (criteria: relative molecular weight molecular weight ≤500 Da, drug-likeness DL ≥0.18) from 4 Chinese medicines (e.g., Panax ginseng, Resina Draconis) contained in the hydrogel and combined them with the GeneCards database to obtain phlebitis-related targets. We predicted transdermal permeability by the Potts-Guy model, constructed protein–protein interaction networks and drug-component-target-disease networks, and conducted gene ontology/KEGG pathway enrichment analysis and molecular docking validation. A total of 90 active ingredients and 221 potential targets were screened, and 21 key targets were obtained after taking the intersection with phlebitis-related targets. Abietic acid, dehydroabietic acid, and oleanolic acid were identified as the core active ingredients, and IL-10, TNF, IL-6, VCAM1, and ICAM1 were identified as the core targets. Enrichment analysis indicated that Qufushengji hydrogel may intervene in immune-inflammatory responses, metabolic complications, and pathogen infection-related mechanisms by modulating AGE-RAGE, TNF, and other pathways. This study reveals the potential mechanism of Qufushengji hydrogel in treating phlebitis through the “multi-component-multi-target-multi-pathway” model, which provides a theoretical basis for the treatment of phlebitis by traditional Chinese medicine.

## 1. Introduction

Intravenous therapy is the most common treatment in the clinic, mainly through peripheral venous catheters or venous cannulation, where the most common adverse effect is phlebitis. Clinically, it presents as localized redness, swelling, heat, pain, and other discomforts of the skin, sometimes accompanied by the appearance of striated lines, and in more serious cases, it will show striated hard nodules and even the appearance of systemic symptoms, including chills and fever.^[1]^ Phlebitis may be caused by clinical infusion of hypertonic, potassium-containing drugs; failure to follow the principles of asepsis; and insertion of large-bore catheters that leads to inflammatory manifestations in the skin of the vessel wall.^[2,3]^

When modern medicine treats phlebitis, topical Western medicine external application has certain efficacy but still has certain limitations, such as the use of magnesium sulfate can promote the swelling of local tissues, but its optimal use may depend on the concentration, temperature, and the combination with other treatments^[4]^; Glycoside alkaloids contained in potatoes can effectively reduce the swelling and alleviate the symptoms of pain,^[5]^ but their quality standards are poorly controllable, the preservation period is difficult to achieve, the production process is poor, and it is not easy to meet the requirements of “safe, effective, stable, and convenient” of traditional Chinese medicine.^[6]^

The Chinese medicine prescriptions we have studied utilize hydrogel as the carrier and Panax notoginseng, Resina Draconis, Bletilla striata and Coptis chinensis as the main ingredients in the formulation. Panax notoginseng can promote blood circulation, improve local blood stasis, and relieve redness and swelling; Resina Draconis has the efficacy of activating blood circulation, removing blood stasis, stopping bleeding, and regenerating muscle; it can improve local blood circulation, reduce redness and swelling, and promote the dissipation of hard, cord-like nodules^[7]^; Bletilla striata is an astringent and hemostatic, reduces swelling and promotes tissue regeneration, helps to repair damaged tissues, promotes the reduction of striated nodules, and reduces tenderness^[8]^; Coptis chinensis can clear heat and dry dampness, expel fire and detoxify, which can effectively inhibit local bacterial growth and reduce inflammation, thus relieving redness, swelling, and tenderness.^[9,10]^ Network pharmacology, as a cutting-edge interdisciplinary discipline, integrates the advantages of systems biology, multi-omics technology, and computer science, providing a new perspective for drug development and mechanism of action interpretation.^[11]^ Different from the traditional pharmacology focusing on the mechanism of a single target, network pharmacology adheres to the concept of “multi-component, multi-target, multi-pathway” and systematically and comprehensively analyzes the complex mechanism of drug intervention in the disease, and this holistic and systematic research idea is highly compatible with the holistic concept of traditional Chinese medicine and the ideology of diagnosis and treatment.^[12]^ From the network pharmacology level, each of these 4 Chinese medicines contains a wide variety of chemical components, each of which can act on multiple different targets, which interact with the key links in the development of phlebitis through complex signaling pathways. Molecular docking can validate the interaction between the active compound and the central therapeutic target.^[13]^ With the help of network pharmacology, we were able to explore the potential molecular mechanism of these 4 Chinese medicines in combating phlebitis, provide rigorous theoretical support for the rational formulation and subsequent validation of the efficacy of these medicines, and open a new pathway for the development of innovative therapeutic strategies for phlebitis based on traditional Chinese medicine.

## 2. Methods

### 2.1. Screening of active compounds and prediction of the targets of Qufushengji hydrogel

Qufushengji hydrogel consists of 4 herbal extracts of Panax notoginseng, Bletilla striata, Coptis chinensis, and Resina Draconis. The Traditional Chinese Medicine Systems Pharmacology (TCMSP, https://old.tcmsp-e.com/tcmsp.php) is a specialized database integrating the chemical composition, pharmacokinetic parameters, and target prediction of traditional Chinese medicines (TCMs), which provides data support for the study of multicomponent mechanisms of action of TCMs.^[14,15]^ High-throughput Experiment- and Reference-guided Database of Traditional Chinese Medicine (HERB, http://herb.ac.cn/) is a high-throughput experiment- and reference-guided database of traditional Chinese medicine, which provides curated compound-target associations supported by experimental evidence.^[16]^ The Herbal Ingredients’ Targets Database (HIT) contains detailed information on interactions between herbal components and their known protein targets based on literature mining.^[17]^ These databases provide comprehensive information on herbal ingredients, pharmacokinetic properties, and target prediction for TCMs. Drug-like properties (DL) reflect the structural similarity of compounds to known drugs, and high DL values (≥0.18) suggest superior pharmacodynamic and pharmacokinetic properties^[18,19]^; Relative Molecular Weight (MW) is a key parameter in the transdermal absorption of topical drugs, and compounds with low MW (≤500 Da) are more likely to penetrate the skin barrier.^[20]^ In conjunction with the TCMSP suggested criteria, the screening of active compounds needs to fulfill DL ≥ 0.18 and MW ≤ 500 Da. Because the TCMSP database did not include Resina Draconis, it was supplemented using the BATMAN-TCM database to include component targets with scores > 40. The BATMAN-TCM database (http://bionet.ncpsb.org.cn/batman-tcm/) is a bioinformatics analysis database for the molecular mechanism of action of traditional Chinese medicine.^[21]^

PubChem, the world’s largest open chemical database, covers structure, activity, and toxicology information for over 100 million compounds.^[22]^ The structures of the compounds were obtained from the PubChem database (https://pubchem.ncbi.nlm.nih.gov), and the compounds were analyzed using the SwissADME platform (http://www.swissadme.ch). To evaluate their absorption, the compounds were screened based on the 5 principles of drug-like properties (Lipinski, Ghose, Veber, Egan, and Muegge). Compounds meeting at least 2 of these criteria^[23]^ were considered to have good bioavailability and drug-like properties and were selected as active ingredients. The active ingredient information obtained from the PubChem database was entered into the SwissTargetPrediction database (http://www.swisstargetprediction.ch), and only the targets with probability >0 were extracted as potential targets. According to the “Probability” value in the target information, only the targets with probability >0 were extracted as potential targets. SwissTarget is based on the principle of ligand similarity and can rapidly predict potential targets and related pathways of small molecule compounds in the human body.^[24]^ UniProt (http://www.uniprot.org/) is the most informative and extensive protein database for standardized gene names.^[25]^ The UniProt database (https://www.uniprot.org/) was used to standardize the target names by entering the protein name. The search was limited to Homo sapiens to remove nonhuman targets. Target proteins were transformed to obtain the corresponding official symbol, and targets without corresponding gene names were deleted.

### 2.2. Collection of gene targets in phlebitis

The GeneCards database (https://www.genecards.org/) is a comprehensive database that can analyze human genetic data. Its main functions include analyzing gene expression, functional pathways, PPIs, and gene-disease relationships.^[26]^ Disease-related targets were searched in the GeneCards database with the keywords “phlebitis” and “superficial phlebitis,” respectively. The results were combined and duplicates were removed. Using the Venn Diagram plotting function of the Bioinformatics and Evolutionary Genomics website (http://bioinformatics.psb.ugent.be/webtools/Venn/), the intersection of the targets of Qufushengji hydrogel and the targets related to phlebitis was obtained, and the Venn Diagram was plotted.

### 2.3. Prediction of transcutaneous drug permeability of key components

The component corresponding to the key target is the key component. Its MW and lipophilicity partition coefficient (log-Kow/AlogP) were checked in the TCMSP database. Atomic Contribution-based logP (AlogP) is a lipid-water partition coefficient calculated based on the atomic contribution method, which is used to predict the partitioning behavior of compounds in octanol-water 2-phase systems, reflecting their lipid solubility strength.^[27]^ The Potts-Guy model is a classical mathematical model for predicting skin permeability coefficient (Kp, cm/s) and logKp in transdermal drug development, which quantifies the transdermal potential of a compound by its physicochemical properties (MW, lipid solubility, hydrogen bonding ability).^[28,29]^ The prediction was performed using the following equation: log KP = 0.71 log-Kow-0.0061MW-6.3.

### 2.4. Protein–protein interaction

Interactions between proteins were predicted using the STRING database (https://string-db.org/). The STRING database system allows for the scoring of forecasts obtained in different ways, with higher scores indicating a higher level of confidence in the protein–protein interaction (PPI) results field.^[30]^ In this study, we utilized the “Multiple Proteins” function of the STRING database, set the “species” as Homo Sapiens, and imported the key targets of Qufushengji hydrogel for phlebitis into the system for searching. The key targets of Qufushengji hydrogel for phlebitis were imported into the system for searching. The PPI network was constructed by setting the score >0.4, and the information reflecting the interrelationship of target proteins, such as the node degree value, was obtained. The obtained PPI network data were imported into Cytoscape 3.10.2 software. Core targets were identified based on degree centrality calculated using the CytoNCA plugin, sort by degree value from high to low,^[31]^ and the top 5 nodes with the highest values were selected. Additionally, layout optimization and node annotation were performed using the AutoAnnotate plugin to enhance clarity.

### 2.5. Network construction

Network construction can screen key nodes from the complex interactions of drugs, target genes, and diseases.^[32]^ Cytoscape 3.10.2 software (http://www.cytoscape.org) was used to construct the drug-component-target-disease network. A node in the network represents Qufushengji hydrogel constituent drugs, drug active ingredients, phlebitis, and its related targets; an edge represents the connection between drug-constituent, constituent-target, and target-disease. The whole network demonstrates the linkage between “drug-component-target-disease,” through which the mechanism of action of intervening diseases can be analyzed in depth. Using network degree and betweenness centrality as screening criteria, the core nodes of the network were obtained through the network connection relationships with these core targets.^[33]^

### 2.6. Gene ontology and Kyoto encyclopedia of genes and genomes pathway enrichment

To gain a deeper understanding of the functions of the obtained core target genes and their roles in signaling pathways, the screened core targets were imported into the platform Metascape (https://metascape.org/gp/index.html), and the corresponding species was set as “Homo Sapiens.” Metascape is a bioinformatics database that integrates biological data and analysis tools to associate genes with biological annotations, identify the most significantly enriched annotation pathways, and help researchers rapidly extract meaningful biological information from large-scale gene fields.^[34]^ Gene ontology (GO) analysis and Kyoto Encyclopedia of Genes and Genomes (KEGG) analysis results were obtained using functional annotation tools in the Metascape database. GO enrichment analysis was used to categorize differentially expressed genes into the 3 main functional categories of GO, namely biological processes (BPs), molecular functions, and cellular components^[35]^; KEGG pathway analysis further revealed the key signaling pathways regulating the pathogenesis of superficial phlebitis, providing a theoretical basis for the exploration of potential therapeutic targets.^[36]^ The top 20 KEGG and top 10 GO results were screened to construct a network for further study.

### 2.7. Molecular docking

The SDF format files of the main active ingredients of the core drugs were obtained through the PubChem database (https://pubchem.ncbi.nlm.nih.gov/), and the key target protein structures were collected in the Protein Data bank database. The Protein Data bank is a global core database for storing 3-dimensional structural data of biological macromolecules, covering structural information of proteins, nucleic acids, complexes, and viruses.^[37]^ Targets were optimized for water molecule and small molecule ligand removal using PyMol-2.1.0 software and hydrogenation and charging using AutoDock Tools-1.5.6 and saved in pdbqt format. Using the key target as the receptor and its corresponding active ingredient as the ligand, molecular docking was performed using Vina-2.0 within the PyRx software to calculate the binding energy and output the result file. The lower the binding energy, the more stable the binding between ligand and receptor.

## 3. Results

### 3.1. Active compounds and targets of Qufushengji hydrogel

With relative MW ≤ 500 Da and DL ≥ 0.18 as the screening criteria, 90 active ingredients and 221 related targets of the drugs were obtained.

### 3.2. Collection of gene targets in Phlebitis

The GeneCards database was searched for disease-related targets using the keywords “phlebitis” and “superficial phlebitis,” and the results were merged and duplicates were removed,resulting in a total of 115 disease targets.

### 3.3. Therapeutic targets of Qufushengji hydrogel for treating Phlebitis

**Figure 1.**
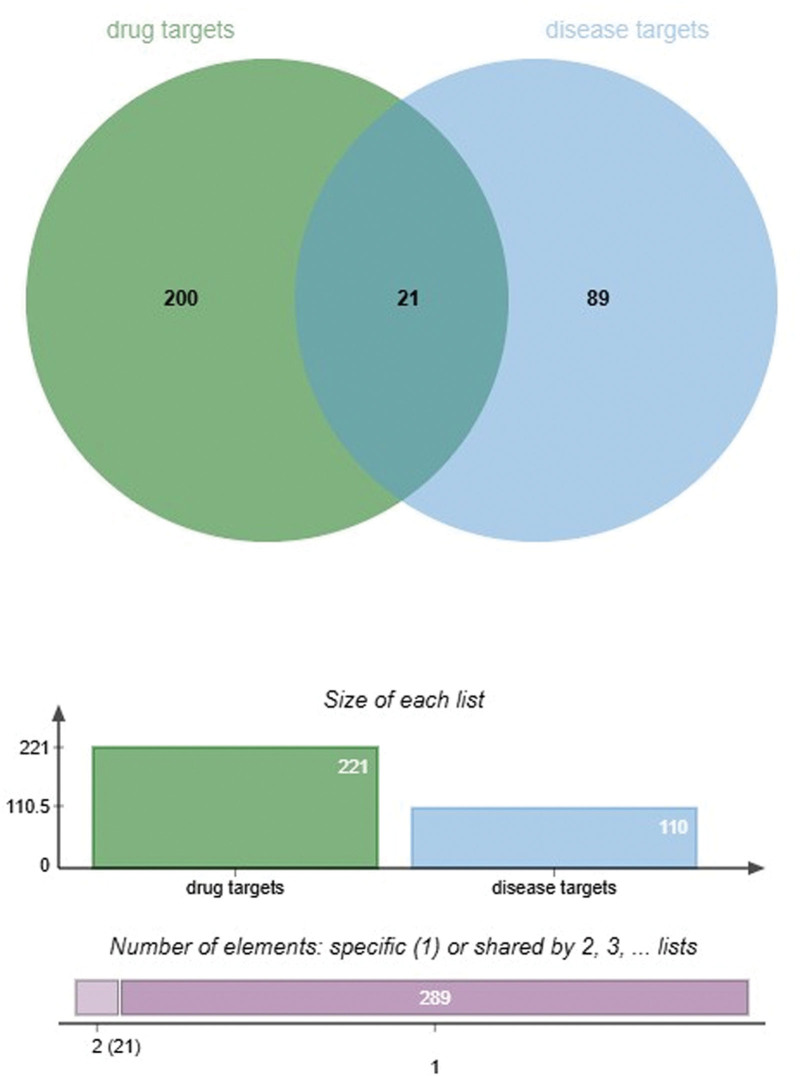
Determination of the overlapping targets of phlebitis and Qufushengji hydrogel. A total of 21 intersecting targets were obtained from Qufushengji hydrogel and phlebitis.

Qufushengji hydrogel and phlebitis had a total of 21 overlapping targets (Fig. 1), which became the focus of subsequent analysis.

### 3.4. Prediction of the transdermal permeability of drugs

Through analysis, 21 key targets originated from 10 active ingredients of Qufushengji hydrogel (i.e., the key ingredients). Their permeability coefficients (Kp, cm/s) were predicted using the Potts-Guy model, and the predicted log Kp values are shown in Table 1.

**Table 1:** Transdermal permeation coefficients of the active ingredients in Qufushengji hydrogel for phlebitis treatment.

	Molecule name	MW	AlogP	Log KP
1	Abietic acid	302.45	5.4	−4.31
2	Dehydroabietic acid	302.42	5.19	−4.46
3	Oleanolic acid	456.78	6.42	−4.53
4	3-(p-hydroxybenzyl)-4-methoxy-9,10-dihydrophenanthrene	348.42	5.11	−4.80
5	4,7-dihydroxy-1-p-hydroxybenzyl-2-methoxy-9,10-dihydrophenanthrene	348.42	5.11	−4.80
6	Palmatine	352.44	3.65	−5.86
7	Columbamine	338.41	3.4	−5.95
8	Physcion	284.28	2.74	−6.09
9	Berlambine	351.38	2.49	−6.68
10	Quercetin	302.25	1.5	−7.08

### 3.5. Therapeutic target PPI network

**Figure 2.**
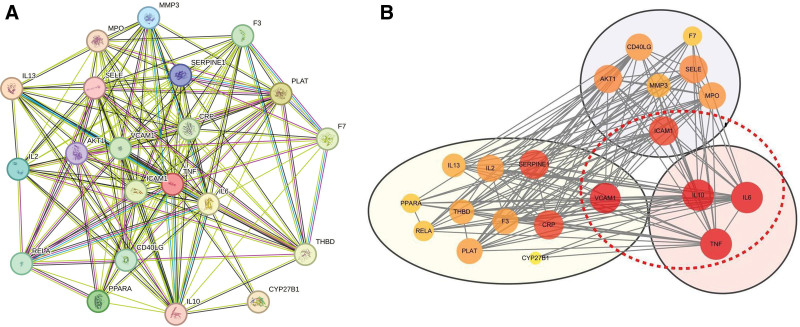
PPI network construction. (A) PPI network of overlapping targets of Qufushengji hydrogel and phlebitis. (B) The core genes of the identified targets. Among all core targets, the deeper the red color and the larger the label, the higher the importance. The core targets are clearly marked with red dotted lines. PPI = protein–protein interaction.

Investigating the network of interactions between proteins can help to uncover coregulated genes. Twenty-one overlapping targets associated with phlebitis and Qufushengji hydrogel were entered into the STRING database to construct the PPI network (see Fig. 2A). The network has 21 nodes and 158 edges with an average degree value of 15. The network was then visualized using Cytoscape 3.10.2. Nodes were sorted by degree in descending order using the CytoNCA plugin. The top 5 targets based on degree value – IL-10, TNF, IL-6, VCAM1, and ICAM1 – were identified as the core targets. To improve the visualization, the AutoAnnotate plugin was used to reduce overlaps and clearly label the core targets (see Fig. 2B).

### 3.6. GO and KEGG pathway enrichment

**Figure 3.**
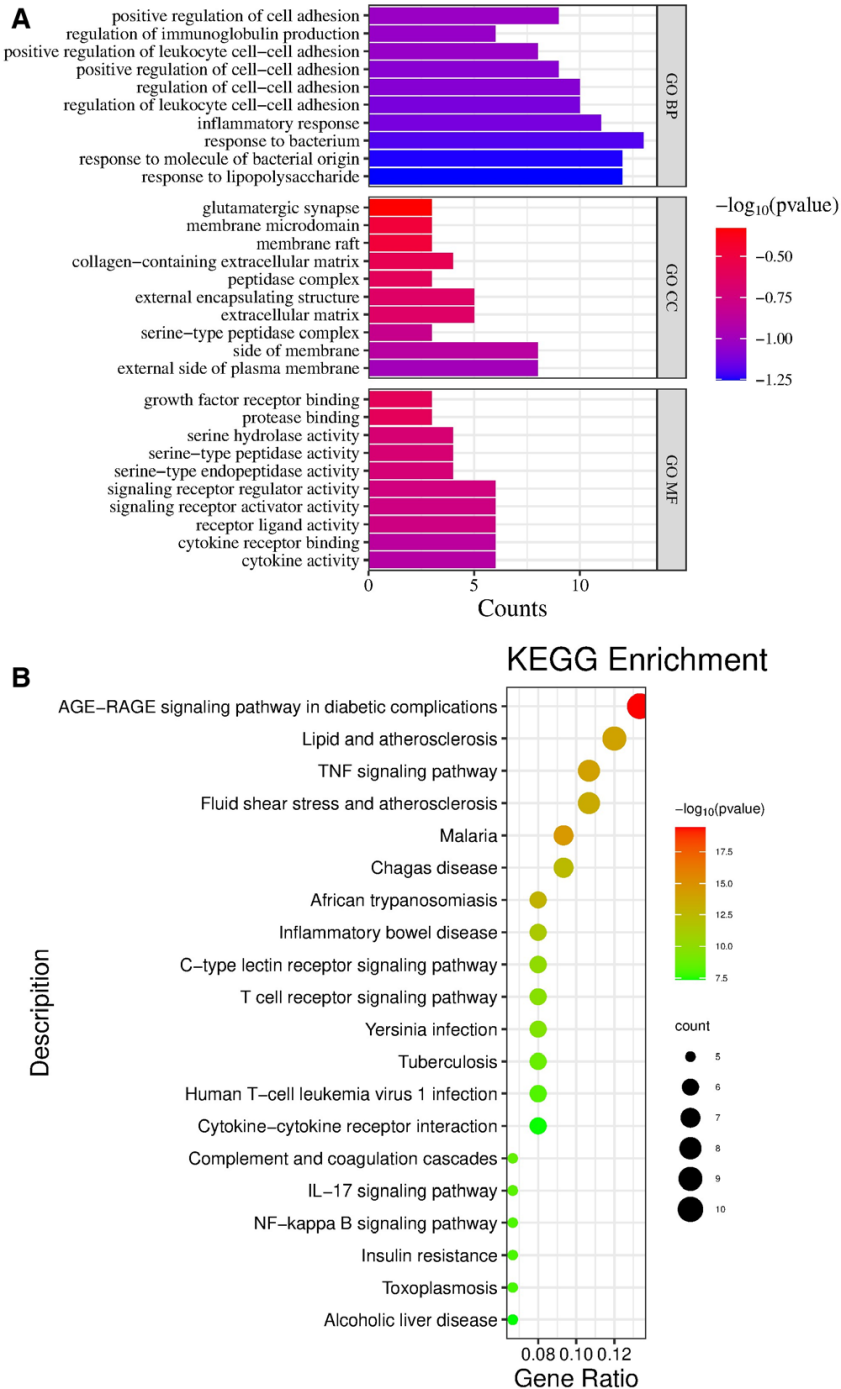
Enrichment analysis results of core targets in Qufushengji hydrogel. (A) Top 10 BP, CC, and MF results in GO annotation analysis. The y-axis stands for enriched GO of the targets, and the x-axis stands for the gene number in the GO annotation results. (B) The top 20 KEGG pathway enrichment bubble diagram (*P* <.05). The y-axis represents the enriched pathways of the targets, and the x-axis represents the values of fold enrichment. BP = biological processes, CC = cellular composition, GO = gene ontology, KEGG = Kyoto encyclopedia of genes and genomes, MF = molecular function.

The 21 overlapping targets were enriched using the Metascape online tool. GO functional enrichment analysis yielded 383 GO results (*P* <.05), including 354 biological process (BP) results, 10 cellular composition (CC) results, and 19 molecular function (MF) results, which accounted for 92%, 3%, and 5% of the results, respectively. KEGG enrichment results screened 75 pathways (*P* <.05). The top 20 KEGG results and the top 10 BP, CC, and MF results in the GO annotation analysis were screened according to the *P* value for visualization, and the results are shown in Figure 3A and B.

As shown in Figure 3A, BP mainly involves positive regulation of cell adhesion, positive regulation of immunoglobulin production, positive regulation of leukocyte cell–cell adhesion, positive regulation of cell–cell adhesion, etc; CC mainly involves glutamatergic synapse, membrane microdomain, membrane raft, collagen-containing extracellular matrix, etc; MF mainly involves growth factor receptor binding, protease binding, serine hydrolase activity, serine-type peptidase activity, etc. This indicates that Qufushengji hydrogel treats phlebitis through multiple mechanisms. As shown in Figure 3B, the genes are significantly enriched in pathways such as the AGE-RAGE signaling pathway in diabetic complications, lipid and atherosclerosis, the TNF signaling pathway, fluid shear stress and atherosclerosis, and Chagas disease. Therefore, it is hypothesized that Qufushengji hydrogel may be beneficial in the treatment of phlebitis by modulating immune and inflammation-related, metabolism and disease complication-related, cardiovascular disease-related, and pathogen infection-related pathways.

### 3.7. Compounds – therapeutic targets network

**Figure 4.**
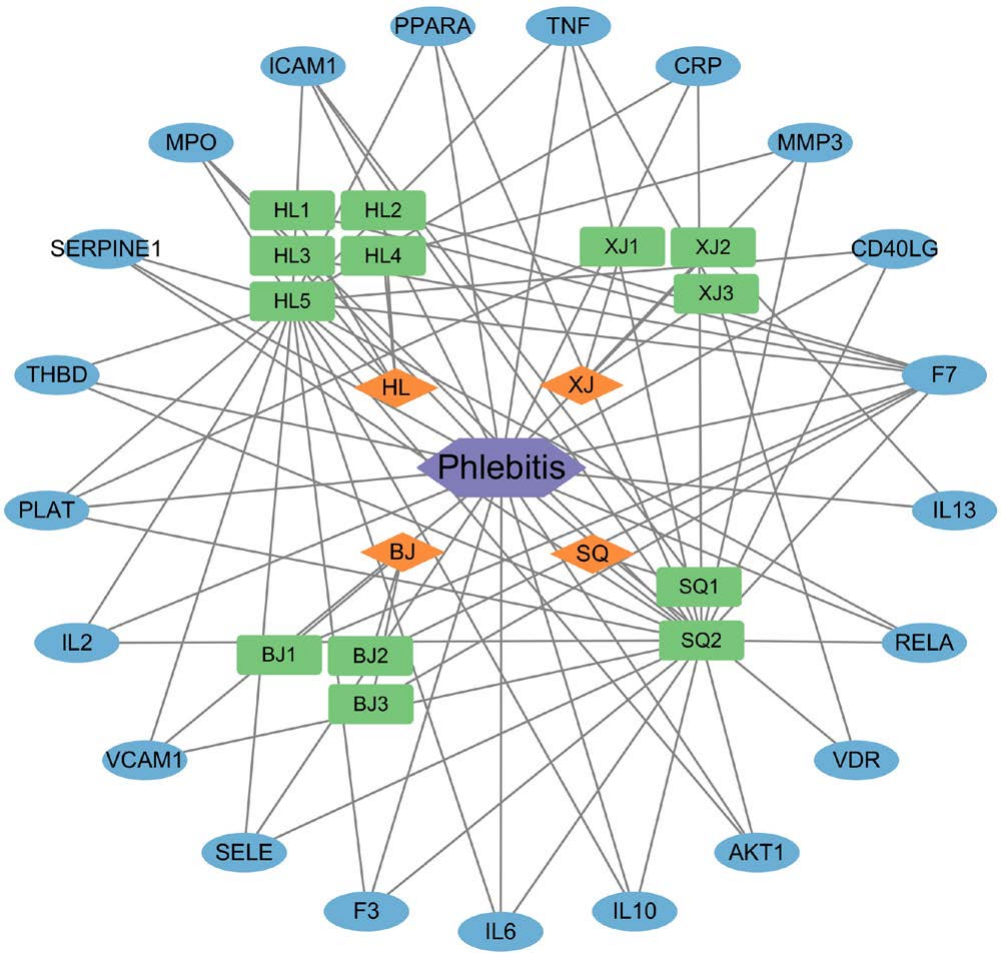
Network construction. The traditional Chinese medicine-compound-target-disease network of core targets between Qufushengji hydrogel and phlebitis. Orange rhombuses represent 4 Chinese herbs in Qufushengji hydrogel, green squares denote major active compounds from these herbs, blue ellipses indicate intersecting targets, and purple hexagons represent phlebitis.

The TCM-compound-target-disease network was mapped using Cytoscape 3.10.2 software (see Fig. 4). The TCM-compound-target-disease network had a total of 39 nodes and 83 edges, and these interactions indicated that 1 compound was able to modulate multiple targets, and 1 target could also be modulated by multiple compounds at the same time.

### 3.8. Results of molecular docking

**Figure 5.**
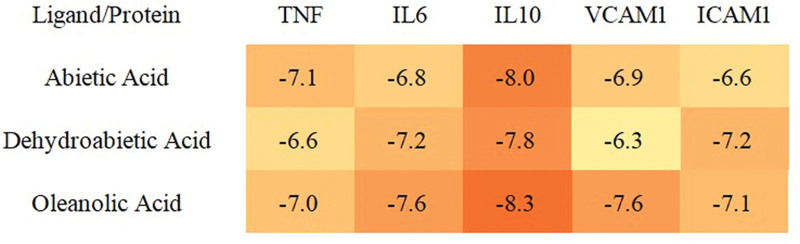
Calculated binding energy values (kcal/mol) for active compound-core target interactions.

**Figure 6.**
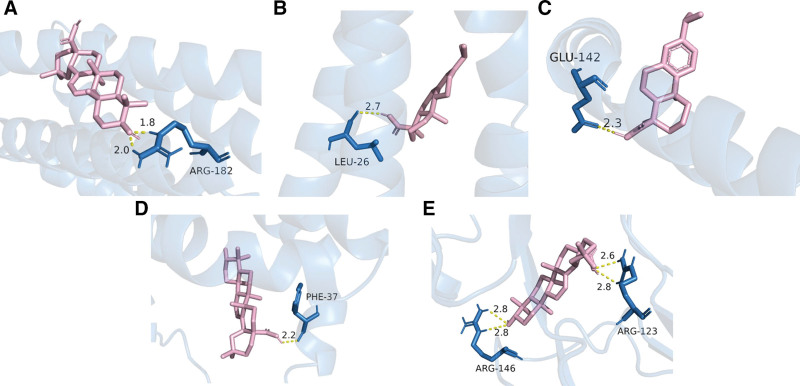
Top 5 compounds bound to the target (pink, compounds; blue, protein). (A) Oleanolic acid and IL-6; (B) abietic acid and IL-10; (C) dehydroabietic acid and IL-10; (D) oleanolic acid and IL-10; (E) oleanolic acid and VCAM1. IL-6 = interleukin-6, IL-10 = interleukin-10, VCAM1 = vascular cell adhesion molecule 1.

Literature studies have reported that the lower the energy at which the ligand-receptor binding conformation is stabilized, the higher the likelihood of interaction. Generally, when the docking fraction value is less than −5 kcal/mol, it indicates that they interact well, and this protein can be a key candidate target for research.^[38]^ The interactions of the 3 key active ingredients with the 5 core targets in the PPI network were evaluated by molecular docking simulations. The simulation results showed that stable binding was formed between these active ingredients and the core targets, and the docking fraction values were all less than −5 kcal/mol, indicating good binding affinity between them. To visualize this interaction more clearly, thermograms of the binding free energy were plotted (see Figs. 5 and 6).

## 4. Discussion

In the present study, we used network pharmacology to investigate the potential active compounds and mechanisms of Qufushengji hydrogel for the treatment of phlebitis. Through screening, we selected 10 active compounds based on their degree of connectivity with the 21 overlapping targets in the compound-target-disease network. After further molecular docking, we identified abietic acid, dehydroabietic acid, and oleanolicacid as the core active ingredients. From the PPI network analysis using CytoNCA plugin, IL-10, TNF, IL-6, VCAM1, and ICAM1 were selected as the core targets of Qufushengji hydrogel, which may play an important role in the treatment of phlebitis.

Additionally, transdermal permeability was evaluated using the Potts-Guy model. The logKp values of the core ingredients such as abietic acid (−4.31) and oleanolic acid (-4.53) suggest moderate skin permeability, which is generally considered favorable for transdermal drug delivery. LogKp values between −2 and −5 indicate acceptable permeability, while values below −6 may result in poor absorption. Thus, several compounds in the hydrogel possess suitable properties for topical application.^[39]^

TNF is a cytokine secreted by macrophages and other cells, which contributes to an increased inflammatory response during phlebitis. Its massive release can activate other inflammatory mediators, leading to endothelial cell damage and localized symptoms of phlebitis, such as redness, swelling, and pain.^[40,41]^ IL-6, an important cytokine, is secreted by a variety of cells during the development of phlebitis. It is involved in the immune response and acute-phase reaction and is able to mobilize more immune cells to the site of phlebitis, exacerbating the inflammatory response and contributing to the progression of phlebitis.^[42]^ IL-10 is a cytokine with immunosuppressive function.^[43]^ In phlebitis, IL-10 can play an anti-inflammatory role, inhibit the production of pro-inflammatory cytokines, reduce the intensity of the inflammatory response, exert a negative regulatory effect on the development of phlebitis, and help to alleviate inflammatory injury. VCAM1 is mainly expressed on the surface of vascular endothelial cells.^[44]^ During phlebitis, its expression is upregulated, mediating the adhesion of leukocytes to vascular endothelial cells. This facilitates leukocyte migration to the site of inflammation, triggering further inflammatory injury and promoting the deterioration of phlebitis. ICAM1 is widely distributed on a variety of cell surfaces. During phlebitis, ICAM1 interacts with integrins on the surface of leukocytes to promote leukocyte adhesion, rolling, and migration, leading to localized aggregation of inflammatory cells in the veins and exacerbating the degree of inflammation in phlebitis.^[45]^ These findings suggest that the effectiveness of Qufushengji hydrogel in the treatment of phlebitis is mainly achieved through the modulation of physiopathological processes related to inflammation and immunity.

Although experimental validation was not performed in this study, numerous existing studies support the relevance of the predicted targets and pathways. For instance, TNF and IL-6 are widely recognized as key cytokines in vascular inflammation and phlebitis progression. TNF promotes leukocyte recruitment and endothelial activation, while IL-6 mediates acute-phase responses and enhances vascular permeability.^[41,42]^ These findings corroborate our network pharmacology predictions.

Regarding the hydrogel’s pharmacological properties, although animal testing was not feasible at this stage, previous literature demonstrates that similar hydrogel formulations exhibit good biocompatibility, minimal irritation, and sustained-release properties suitable for transdermal drug delivery.^[46]^ These references provide indirect evidence supporting the safety and applicability of our hydrogel system.

In an acute superficial thrombophlebitis rabbit model, topical administration of a herbal phlebitis ointment significantly reduced TNF‑α and IL‑6 levels compared to a positive control group, and showed improved histopathological outcomes.^[47]^ This suggests that TCM formulations may provide stronger anti-inflammatory effects than conventional agents. These findings highlight the clinical potential and multi-target advantages of herbal-based therapies.

GO and KEGG enrichment analyses were performed on the intersecting targets. According to the GO analysis, the intersecting targets showed strong correlations with BP (positive regulation of cell adhesion, positive regulation of immunoglobulin production, positive regulation of leukocyte cell–cell adhesion), CC (glutamatergic synapse, membrane microdomain, membrane raft), and MF (growth factor receptor binding, protease binding, serine hydrolase activity). Therefore, Qufushengji hydrogel may exert its effects through the above pathways. According to the KEGG analysis, the therapeutic targets of Qufushengji hydrogel for phlebitis are mainly associated with pathways such as the AGE-RAGE signaling pathway in diabetic complications, Lipid and atherosclerosis, the TNF signaling pathway, and Fluid shear stress and atherosclerosis.

In terms of future research, animal experiments are warranted to validate the anti-inflammatory efficacy of Qufushengji hydrogel, particularly its regulatory effects on TNF, IL-6, and VCAM1 pathways.^[48]^ Moreover, the pharmacokinetics and sustained-release behavior of the hydrogel should be optimized to improve clinical efficacy and bioavailability.^[46]^

## 5. Conclusion

In this study, the multidimensional mechanism of action of Qufushengji hydrogel for the treatment of phlebitis was systematically revealed by network pharmacology and molecular docking technology. The compound acts on 21 core targets through 10 key active ingredients (including oleanolic acid andabietic acid) and exerts synergistic therapeutic effects via AGE-RAGE, TNF, and other signaling pathway networks. The pivotal targets, such as IL-6, TNF, IL-10, VCAM1, and ICAM1, improve the pathological state of phlebitis through the modulation of inflammatory mediator release, vascular endothelial repair, and immune regulation. Molecular docking demonstrated that the active ingredients have strong binding affinity to these targets. Future studies should focus on verifying pathway activity through animal experiments and optimizing the hydrogel’s pharmacokinetic and release properties to enhance clinical translation.

## Acknowledgments

The authors would like to thank all the staff who participated in this study, as well as the Jilin Provincial Department of Science and Technology for its financial support (project number: YDZJ202601ZYTS725).

## Author contributions

**Conceptualization:** Zhe Meng, Xiuling Zhou.

**Data curation:** Shuangxin Zhang, Qirui Zhang, Yijia Lin.

**Formal analysis:** Juncai Li, Longjie Wei, Bonolo William.

**Supervision:** Xiuling Zhou.

**Writing – original draft:** Zhe Meng.

**Writing – review & editing:** Xiuling Zhou.
